# CARE PRIORITIES AND PREFERENCES OF PEOPLE LIVING WITH DEMENTIA DURING EMERGENCY DEPARTMENT VISITS

**DOI:** 10.1093/geroni/igae098.2581

**Published:** 2024-12-31

**Authors:** Clark Benson, Kristin Merss, Laura Vergenz, Kayla Dillon, Laura Block, Andrea Gilmore-Bykovskyi

**Affiliations:** University of Wisconsin School of Medicine and Public Health - Madison, WI, Madison, Wisconsin, United States; University of Wisconsin Madison, Madison, Wisconsin, United States; University of Wisconsin School of Medicine and Public Health - Madison, WI, Madison, Wisconsin, United States; University of Wisconsin School of Medicine and Public Health - Madison, WI, Madison, Wisconsin, United States; University of Wisconsin-Madison, Madison, Wisconsin, United States; University of Wisconsin Madison, Madison, Wisconsin, United States

## Abstract

Over half of the 6 million persons living with dementia (PLWD) in the United States visit an Emergency Department (ED) annually. Dementia is often under-recognized in the ED, and visits are associated with significant adverse outcomes for PLWD. Yet, little is known about the perspective and priorities of PLWD and their caregivers regarding ED care. In this descriptive qualitative study, we examined the priorities of PLWD and caregivers during ED visits. We recruited patients living with dementia from a large academic ED with options to participate in a semi-structured interview during or after their ED visit and individually or dyadically with a caregiver. All PLWD completed capacity assessments prior to the interview. Capacity assessments were completed for all PLWD participants. We iteratively analyzed interviews using thematic analysis. We conducted interviews with 33 participants (N=10 PLWD, 15 caregivers, 4 dyads). PLWD and caregivers largely evaluated the ED experience positively. Findings highlight aspects of ED care uniformly shared among participants, including being informed at every step. Our findings also describe individually specific values and preferences such as approach to decision-making; as well as conditions that shape priorities across the experience such as degree of uncertainty. Both PLWD and caregivers value feeling informed in the ED and cite the importance of awareness of the impact of cognition on care. Our findings will support the development of patient-centered assessment tools to evaluate PLWD ED care experiences, enabling proximal and sensitive evaluation of within ED care practices for this vulnerable population.

